# The Management of Dry Eye Disease: Proceedings of Italian Dry Eye Consensus Group Using the Delphi Method

**DOI:** 10.3390/jcm11216437

**Published:** 2022-10-30

**Authors:** Pasquale Aragona, Giuseppe Giannaccare, Rita Mencucci, Pierangela Rubino, Emilia Cantera, Claudia Yvonne Finocchiaro, Sabrina Vaccaro, Francesco Aiello, Elena Antoniazzi, Stefano Barabino, Stefano Bonini, Gianpaolo Carlini, Chiara Chierego, Rossella Anna Maria Colabelli Gisoldi, Antonio Di Zazzo, Romina Fasciani, Antonella Franch, Giovanna Gabbriellini, Caterina Gagliano, Andrea Leonardi, Angelo Macrì, Luigi Mosca, Vincenzo Orfeo, Antonio Pinna, Augusto Pocobelli, Romolo Protti, Paolo Rama, Laura Rania, Miguel Rechichi, Andrea Russo, Vincenzo Scorcia, Leopoldo Spadea, Marco Trentadue, Salvatore Troisi, Piera Versura, Edoardo Villani, Maurizio Rolando

**Affiliations:** 1 Department of Biomedical Sciences, Ophthalmology Clinic, University of Messina, 98121 Messina, Italy; 2 Department of Ophthalmology, University “Magna Græcia” of Catanzaro, Viale Europa, 88100 Catanzaro, Italy; 3 Eye Clinic, Department of Neuroscience, Psychology, Pharmacology and Child Health, University of Florence, 50134 Florence, Italy; 4 Ophthalmology Unit, Department of General and Specialized Surgery, University Hospital of Parma, Via Gramsci 14, 43126 Parma, Italy; 5 Israelitic Hospital, 00148 Rome, Italy; 6 Private Psychologist, 20100 Milan, Italy; 7 Ophthalmology Unit, Department of Experimental Medicine, University of Rome Tor Vergata, 00173 Rome, Italy; 8 Foundation IRCCS Policlinico San Matteo, 27100 Pavia, Italy; 9 Ocular Surface and Dry Eye Center, Ospedale L. Sacco, University of Milan, 20157 Milan, Italy; 10 Department of Ophthalmology, University of Rome Campus Biomedico, 00128 Rome, Italy; 11 ASL Napoli 1 Centro, 80145 Napoli, Italy; 12 Ophthalmic Unit, Department of Neurosciences, Biomedicine and Movement Sciences, University of Verona, 37129 Verona, Italy; 13 San Giovanni Addolorata Hospital, UOC Oftalmologia-Banca degli Occhi, 00184 Rome, Italy; 14 Fondazione Policlinico Universitario A. Gemelli-IRCCS, 00168 Rome, Italy; 15 Fondazione Banca Degli Occhi del Veneto Onlus, Department of Ophthalmology, SS Giovanni and Paolo Hospital, 30122 Venice, Italy; 16 Ophthalmology, Department of Surgical Medical, Molecular Pathology and of the Critical Area, University of Pisa, 56126 Pisa, Italy; 17 Ophthalmology Clinic, San Marco Hospital, University of Catania, 95123 Catania, Italy; 18 Ophthalmology Unit, Department of Neuroscience, University of Padua, 35121 Padua, Italy; 19 IRCCS San Martino Polyclinic Hospital, 16132 Genoa, Italy; 20 Ophthalmology Unit “Clinica Mediterranea”, 80122 Naples, Italy; 21 Department of Medicine, Surgery and Pharmacy, Ophthalmology Unit, University of Sassari, 07100 Sassari, Italy; 22 Ophthalmic Unit, Hospital San Biagio, 28845 Domodossola, Italy; 23 Cornea and Ocular Surface Unit, San Raffaele Scientific Institute, 20132 Milan, Italy; 24 Department of Ophthalmology, Istituto per la Sicurezza Sociale, San Marino State Hospital, 387261 Cailungo, San Marino; 25 Centro Polispecialistico Mediterraneo, 88050 Selia Marina, Italy; 26 Eye Clinic, Department of Neurological and Vision Sciences, University of Brescia, Piazzale Spedale Civili, 1, 25100 Brescia, Italy; 27 Eye Clinic, Policlinico Umberto I, “Sapienza” University of Rome, 00161 Rome, Italy; 28 Azienda Ospedaliera “Ospedale Consorziale Policlinico”, 70124 Bari, Italy; 29 Eye Department, AOU “San Giovanni di Dio e Ruggi d’Aragona”, 84131 Salerno, Italy; 30 Ophthalmology Unit, Department of Experimental, Diagnostic and Specialty Medicine (DIMES), Alma Mater Studiorum University of Bologna, S. Orsola-Malpighi Teaching Hospital Bologna, 40138 Bologna, Italy; 31 Eye Clinic, San Giuseppe Hospital, IRCCS Multimedica, University of Milan, 20123 Milan, Italy; 32 Ocular Surface and Dry Eye Center, ISPRE Ophthalmics, 16129 Genoa, Italy

## 1. Introduction

Dry eye disease (DED) is a highly prevalent, chronic and progressive condition that affects 5–33% of the world’s adult population [1]. The 1995 definition of DED only considered patient-reported symptoms (ocular discomfort) and damage to the inter-palpebral ocular surface [2]. However, as it became apparent that this failed to reflect the complexity of the disease and its impact on visual function, inducing a risk of under-diagnosis, the 2007 International Dry Eye WorkShop (DEWS) redefined it as follows: “*A multifactorial disease of the tears and ocular surface that results in symptoms of discomfort, visual disturbance, and tear film instability with potential damage to the ocular surface. It is accompanied by increased osmolarity of the tear film and inflammation of the ocular surface*” [3]. This introduced the concept that the ocular surface is a single system, added visual disturbances to the symptoms of ocular discomfort and drew attention to the key concepts of inflammation and tear hyperosmolarity.

Subsequently, as it is not unusual in everyday clinical practice to encounter patients with moderate–severe symptoms who have no pathological signs on the ocular surface or, conversely, patients with severe signs who are asymptomatic because of decreased corneal sensitivity, DEWS II revised its definition to read *“Dry eye is a multifactorial disease of the ocular surface characterized by a loss of homeostasis of the tear film, and accompanied by ocular symptoms, in which tear film instability and hyperosmolarity, ocular surface inflammation and damage, and neurosensory abnormalities play etiological roles”* [4] in order to indicate the occurrence of corneal nerves impairment, too. 

The symptoms characterizing the disease can severely affect the patients’ quality of life and everyday activities such as reading, driving or working on a computer [5,6,7,8] and are also associated with high levels of anxiety and depression [9]. Consequently, it is not only important to prescribe the appropriate treatment, but also to monitor its effects over time in order to ensure long-term relief and prevent disease chronicity [10,11].

Clinicians are clearly aware of the need to adopt a standardized approach to diagnose and treat DED that includes counselling, patient education and the establishment of a medical alliance to promote effective treatment [12,13].

The aim of this paper is to describe the process used by a group of Italian ophthalmologists (“Italian Dry Eye Consensus Group”) focused on DED for identifying four major statements related to the disease aimed at improving overall DED patient care [14]. Given the complexity of the disease and the different clinical contexts in which it may occur, the method used was based on real-life experience, as well as scientific data, and allowed the consideration of areas of still uncertain or unproven knowledge that may nevertheless help to guide everyday clinical practice and future research.

## 2. Methods

Health professionals face the problem of having to reach decisions even when there is insufficient information or conflicting data. Statistical methods such as meta-analyses are very effective in summarizing and comparing the results of clinical trials, but they can sometimes be difficult to transfer to real life. Consequently, we chose to use a consensus meeting format based on the Delphi method [1,15] in order to combine published scientific data with the first-hand experience of Italian ophthalmologists, determining the extent to which experts agree on a particular question and obtain shared confirmation of their opinions [15,16,17].

The project was divided into 4 phases, the first of which involved a group of 5 Italian experts in ocular surface diseases (P.A., E.C., R.M., P.R., M.R.—P.I.C.A.S.S.O. board members) who had previously collaborated in defining a standard approach to DED prevention and treatment [13]. On the basis of their analyses of the literature, they defined 4 areas of consideration: (1) managing DED and inflammation over time; (2) managing eyelid disorders in patients with DED; (3) managing the ocular surface in surgical patients; and (4) improving patient satisfaction, collaboration and treatment adherence. 

The second phase involved 35 highly qualified opinion leaders from the entire territory of Italy who were asked to review a selection of articles published by the members of the P.I.C.A.S.S.O. group [6,7,10,12,18,19,20,21,22,23,24,25,26,27,28,29,30,31,32,33,34,35,36,37,38] and answered an initial round of questions in the light of their clinical experience by ranking 4 proposed responses. The questions were answered independently and anonymously using an online portal, and the participants had no access to the answers of the other clinicians. Subsequently, without being able to see the results of the analysis of the answers to the first round of questions (in order to keep them masked about the answers of the other group members) or their own previous answers, they were asked to evaluate each of the proposed answers to the questions asked in the first round using a 1–5 Likert scale (1 = complete disagreement and 5 = complete agreement). Once again, their responses were entered anonymously, and they had no access to the responses of the other clinicians.

In the third phase, the 35 clinicians joined an in-person meeting on 17 May 2019 to discuss the results of the 2 rounds of questions (presented in statistical form) with the aim of formulating statements that they could agree upon. The meeting was moderated by a psychologist (C.Y.F.), who allowed everyone to speak and ensured the fair distribution of the time allocated to each subject. The members were encouraged to express their opinions freely and enrich the discussion with reflections, criticisms, objections and doubts based on their personal experience. At the end of each discussion, a statement was proposed by a communication expert who had been present throughout the meeting, and the clinicians could propose changes and/or additions on the basis of common agreement. Each of these discussions was allocated the same amount of time and led to the definition of the final version of the statement concerned.

In the fourth phase, each statement was read aloud and projected on a screen, and the clinicians were asked to indicate whether they agreed with it or not. If agreement was ≥80%, it was to be considered accepted; if it was less, the statement could be further revised and submitted to a second and final vote. 

## 3. Results

All of the experts involved answered all of the questions, and there were no abstentions. All four statements were accepted without the need for a second vote.

The following paragraphs describe the quantitative data relating to the second rounds of questions, the qualitative data arising from the plenary discussions that led to the creation of the statements and the results of the final vote of acceptance.

### 3.1. Managing Dry Eye Disease and Inflammation over Time

**Figure 1:**
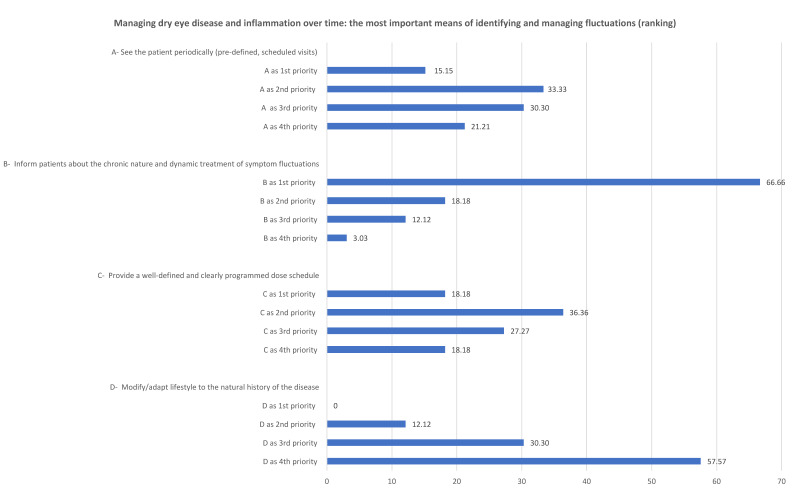
The ranking of the priorities attributed to the two statements related to managing dry eye disease and inflammation over time.

Most of the clinicians felt that the most important aspect of recognizing and treating the fluctuations of DED symptoms is to inform patients about the chronic nature and dynamic treatment of the disease. Figure 1 shows the results of the first round of questions. 

The Likert scale scores obtained from the second round of questions show that the clinicians agreed with all of the proposed means of optimizing the management of DED. They were totally unanimous in agreeing with the importance of informing the patients about the chronic nature and dynamic treatment of symptom fluctuations (100%) and only slightly less than unanimous in agreeing with the need to see patients regularly (97%), to provide a well-defined and programmed dose schedule (95%) and to modify lifestyle in accordance with the natural history of the disease (93%).

After the presentation of the quantitative data arising from the two rounds of questions, the qualitative discussion led to 97.1% acceptance of the first statement: *“The most important aspect for recognizing and treating signs and symptoms of dry eye disease is educating patients about its chronic nature. Furthermore, it should be stressed the need for a dynamic treatment by proposing a programmed dose schedule that also takes into account patients’ lifestyle.”*

The importance of communication and clinician–patient relationships was repeatedly highlighted during the discussion because, in order to be able to recognize and treat the signs and symptoms of DED, it is necessary to inform patients about the disease (particularly its chronic nature) and the continuous and dynamic treatment required to manage its fluctuating symptoms. However, as it was acknowledged that in everyday clinical practice, too little structured time is dedicated to patient education, it was suggested that consideration should be given to the opportunity to dedicate an appropriately trained assistant who could handle this aspect of patient management (as in the case of other diseases) because this would benefit patients and clinicians alike.

It was pointed out that once patients have been educated about the disease, it is important to establish a planned therapeutic regimen rather than leaving treatment decisions up to the patients themselves. This requires clearly defining a specific treatment regimen and dosing schedule that is in line with the patient’s lifestyle and based on the residence time of the tear substitute over the ocular surface.

Given that patients usually do not understand that DED is a chronic condition and are inclined to underestimate the importance of treatment, it was stressed that diminutive words such as “droplets” should be avoided in favor of more appropriate terms such as “tear substitute” or “artificial tear”. The posology regimen “as needed” was also criticized because it suggests that patients can autonomously decide when a symptom should be treated, whereas there is a need for continuous ocular surface nutrition regardless of patient’s ocular discomfort [39]. It was also considered important to explain that treatment is not prescribed to treat the perceived symptom but in order to treat the underlying cause of the symptom.

The third subject of discussion was the importance of not neglecting the patient’s lifestyle, which needs to be taken into account when prescribing treatment and establishing an appropriate dose schedule. It was also pointed out that treatment should be adapted to the environment in which patients live, to the work type and place, to the diet and also to the seasonality. 

It was finally suggested that doctor–patient communications could be improved by means of apps that ask patients questions about their symptoms, treatment compliance and the features of the disease over time.

### 3.2. Managing Eyelid Disorders in Dry Eye Disease

**Figure 2:**
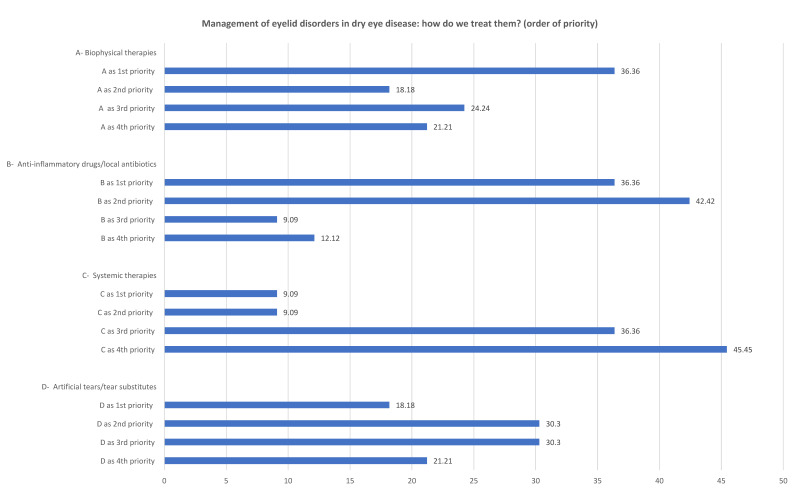
The ranking of the priorities attributed to the two statements related to the management of eyelid disorder in dry eye disease.

To treat the eyelid disorders associated with DED, the clinicians considered eyelid therapies (e.g., hygiene) and anti-inflammatory medications/topical antibiotics equally important (both were ranked first priority by 36.3% of the clinicians), whereas systemic therapies were at the bottom of the list. Figure 2 shows the results of the first round of questions.

The second round of questions showed that clinicians were more in favor of eyelid treatments (42.9% fully agreed, 17.9% agreed and 25.0% agreed with reservations) and anti-inflammatory drugs/topical antibiotics (45% fully agreed, 25.8% agreed and 22% agreed with reservations) rather than systemic therapies (30% fully agreed and 53.3% agreed).

The plenary discussion after the presentation of the quantitative results led to 100% acceptance of the second statement: “Problems of the eyelid margin that are not of surgical interest may cause and/or exacerbate dry eye disease; they should be treated with hygienic treatments, topical corticosteroid medications, specific topical and/or systemic antibiotics, and tear substitutes in a personalized manner, depending on the nature of the change in the eyelid margin and ocular surface, the stage of the disease, and the type of patient.” 

The wording of the statement reflects the complexity of the treatment. The clinicians first discussed their lack of confidence in self-managing systemic therapy and pointed out the importance of educating patients about hygienic treatments and the need to use them when the meibomian glands are still present, before the eventual progression towards an atrophic process, when any therapeutic action is likely to be useless. 

One point that recurred throughout the discussion was the complexity of eyelid treatments in the context of DED. As there are no universal data in the literature, real-life working experience is valid when making decisions about a particular clinical case.

### 3.3. Managing the Ocular Surface in Surgical Patients

**Figure 3:**
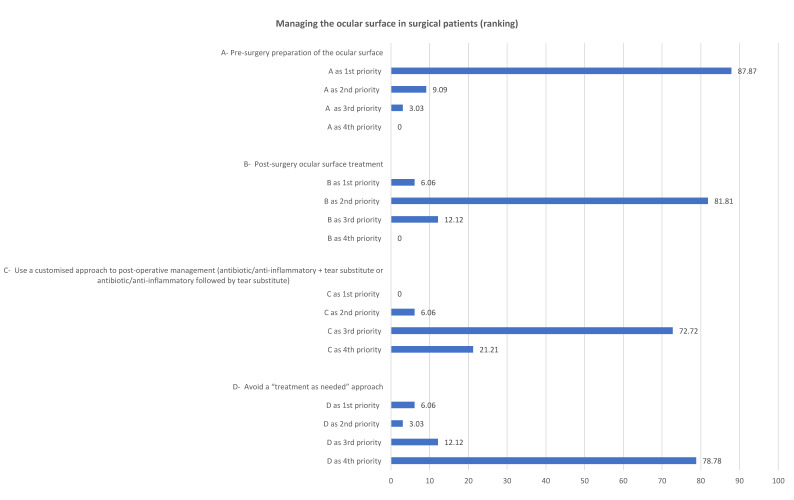
The ranking of the priorities attributed to the two statements related to managing the ocular surface in surgical patients.

Regarding the ocular surface treatment in patients undergoing eye surgery, 87.8% of the clinicians considered preoperative ocular surface optimization the first priority, followed by postoperative ocular surface treatment. Figure 3 shows the results of the first round of questions. 

The results of the second round confirmed the data obtained from the first round. Most of the clinicians (67.7%) fully agreed with presurgical ocular surface optimization, 29% agreed and only 3.2% agreed with reservations. They also agreed that the ocular surface should be treated after surgery (64.5% fully agreed and 32.3% agreed) and that a “treatment as needed” approach should be avoided (61.3% fully agreed, 25.8% agreed and 9.7% agreed with reservations). However, only 16.1% fully agreed that postsurgical treatment should include ocular surface therapy (antibiotic/corticosteroid combination + tear substitute or antibiotic/corticosteroid combination followed by tear substitute), while 41.9% agreed and 35.5% agreed with reservations.

The plenary discussion based on the quantitative results led to the formulation of the third statement with 100% acceptance: “Ocular surface abnormalities may be a risk factor for poorer surgical outcomes and postoperative adverse events. It is important to evaluate and treat the ocular surface before, during and after surgery in order to optimize surgical outcomes”.

The first aspect that was emphasized by the clinicians was the importance of a thorough pre-surgery examination aimed at detecting any changes in the ocular surface that may be a risk factor for a worse outcome and/or post-surgery adverse events. They stressed the importance of informing patients of the presence of any preoperative DED signs so that any eventual postoperative problem is not considered a direct consequence of the surgery. In addition, they agreed on the importance of treating DED before surgery and, if necessary, postponing the operation until the treatment is successful and the ocular surface disease well controlled. 

It was also agreed that the ocular surface should also be examined and treated after surgery and that, in some cases, the surgical technique (e.g., the location of corneal incisions) may be modified in order to better protect the ocular surface. 

Finally, it was pointed out that in real-world clinical practice, organizational and/or bureaucratic issues often limit the time available to assess the ocular surface of patients undergoing surgery and that there is no consensus about the time interval required between the preoperative ocular surface assessment and the day of the operation.

### 3.4. Improving Patient Satisfaction, Collaboration and Treatment Adherence

**Figure 4:**
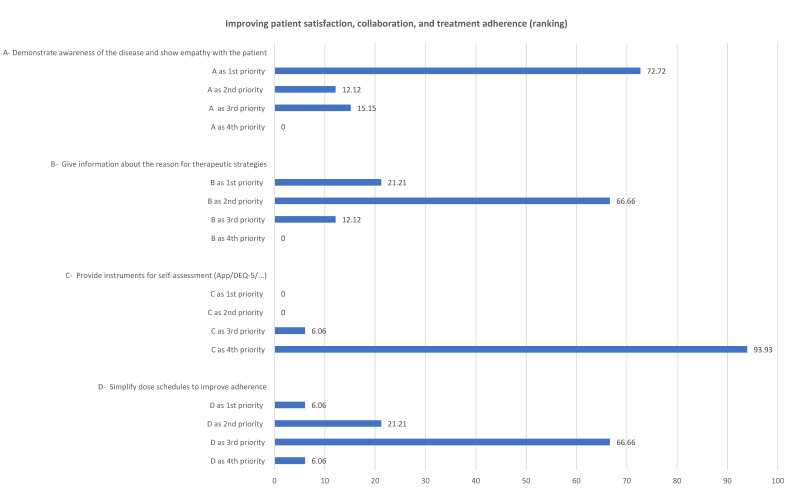
The ranking of the priorities attributed to the two statements related to improving patient satisfaction, collaboration and treatment adherence.

In order to improve patient satisfaction, collaboration and treatment adherence, 72.7% of the clinicians indicated that it was most important to demonstrate an awareness of the disease and show empathy with the patient. Figure 4 shows the results of the first round of questions.

The majority of the clinicians reiterated this point of view in the second round of questions (56.7% fully agreed, and the remaining 45.3% agreed) and also supported the idea of giving more detailed information about the reason(s) for which a particular therapeutic strategy was chosen (45.2% fully agreed, 41.9% agreed and 9.7% agreed with reservations), simplifying dosing regimens to improve adherence (33.3% fully agreed, 57.7% agreed and 3.3% agreed with reservations) and developing technologies that would better achieve patient well-being over time (9.7% fully agreed, 48.4% agreed and 25.8% agreed with reservations).

The plenary discussion based on the quantitative results led 96.8% acceptance of the fourth statement: “Empathy and dialogue are essential for the optimal management of dry eye disease, as is patient treatment adherence in the context of a true therapeutic alliance. Self-assessment technologies and instruments could simplify treatment and improve outcomes.”

The discussion emphasized the need to recognize the importance of patient–physician relationships and a shared therapeutic alliance in ensuring treatment adherence and providing a real benefit for patients. This requires dialogue and an empathic relationship that makes patients feel cared for. The subjective nature of the symptoms associated with DED means that physicians need to understand the condition of their patients not only in terms of the objective features of their eyes but also in terms of their personal space. The clinicians pointed out that they often deal with depressed or anxious patients who have a reduced quality of life, and it is not always clear whether their psychological symptoms are themselves the cause or the consequence of DED. In these circumstances, it is important to take sufficient time to clearly explain the features of this chronic condition and its fluctuating course and avoid creating unrealistic expectations. It was also pointed out that it is possible to diagnose DED quickly and that the remaining time during the visit should be dedicated to explaining the disease and its treatment in order to establish a trusting relationship; however, it is important to remember that patients should be given at least the same time to speak as that taken up by the clinician’s explanations.

The discussion also considered the use of technologies such as apps as a tool of improving disease management. Some of the clinicians said that they were having a hard time adapting to new technological developments but were beginning to think about how they could be profitably used by them and their patients: e.g., to capture a patient’s subjective feelings, to remind patients when to administer the treatment, or to monitor patients’ status over time. Some pointed out that these technologies should not be used to put patients in direct contact with their physicians but to provide summary reports of collected patient data that can be given to a specialist at the time of a visit, thus creating a computerized clinical diary that can be used to track the fluctuations in the disease as it becomes chronic. 

All of the clinicians agreed that technology could not replace an empathic medical relationship or diminish the importance of long-term follow-up, but it could be used to facilitate both these aspects. Although it is important to look for objective signs of disease, some of the clinicians said it was also important to assess psychological variables, as DED is often associated with depressive syndromes. Such assessments could be made as part of a patient interview but also by using self-completed questionnaires that evaluate patient depression and anxiety scores as well as their quality of life. This may not only improve patient’s subjective perceptions but also allows monitoring the effects of treatment on symptoms and the patients’ subjectively perceived quality of life.

Finally, the clinicians considered the economic burden of treatment (which is currently entirely covered by patients in Italy) and expressed the hope that the National Health Service would finally recognize DED as a real disease, thus allowing the reimbursement of its costs.

## 4. Discussion 

The collaborative work of the 35 Italian clinicians led to the definition of four statements using a method that, although it cannot replace the scientific rigor of a systematic review or meta-analysis, allows the consideration of real-life experiences and the applicability of certain guidelines in specific reference contexts.

It is clear that DED not only affects tear film but also the ocular surface as a complex whole. It therefore requires appropriate diagnosis and treatment, which should be adapted to each individual patient on the basis of the predominant disease mechanism (inflammation, epithelial damage or tear film instability) while also taking into account the condition of the eyelids and corneal nerves [12]. Given the chronic and fluctuating nature of the disease and its treatment, communicating with and educating patients are essential means of helping patients understand that treatment must be dynamic and modified over time in order to adapt to changes in the clinical picture [12]. Our experts also emphasized the importance of jointly developing a treatment strategy with patients that takes into account environmental factors, lifestyle, working activities and also seasonal factors [18,21,24,37,38].

It was also mentioned that, in selected cases, it is possible to use technology (e.g., apps) as a tool of subjectively and objectively recording patient symptoms and signs and keeping a structured diary to monitor the situation over time. 

As symptoms are subjective and may not correspond to the objective picture of the disease, it is important to establish good, empathic doctor–patient relationships that allow consideration of the patients’ quality of life—a clinical outcome that should have the same dignity as improving objective signs. Good treatment adherence requires clinicians to prescribe (and not suggest) medications and give clear instructions about how, when and how frequently to administer them [32].

After the consensus conference, the clinicians involved continued to pursue the project by organizing local regional courses where they described the statements and their characteristics and addressed the question of communication and relationship styles in the context of patient management. The work of the expert group also continued with a series of interactive discussions aimed at creating algorithms that are useful in clinical practice, and it is hoped that they will also contribute to further research into dry eye disease.

## References

[1] (2007). Research in dry eye: Report of the research subcommittee of the International Dry Eye WorkShop (2007). Ocul. Surf..

[2] Lemp M.A. (1995). Report of the National Eye Institute/Industry workshop on clinical trials in dry eyes. CLAO J..

[3] (2007). 2007 Report of the International Dry Eye WorkShop (DEWS). Ocul. Surf..

[4] Craig J.P., Nichols K.K., Akpek E.K., Caffery B., Dua H.S., Joo C.K., Liu Z., Nelson J.D., Nichols J.J., Tsubota K. (2017). TFOS DEWS II definition and classification report. Ocul. Surf..

[5] Nichols K.K. (2006). Patient-reported symptoms in dry dye disease. Ocul. Surf..

[6] Friedman N.J. (2010). Impact of dry eye disease and treatment on quality of life. Curr. Opin. Ophthalmol..

[7] Grubbs J.R., Tolleson-Rinehart S., Huynh K., Davis R.M. (2014). A review of quality of life measures in dry eye questionnaires. Cornea.

[8] Miljanović B., Dana R., Sullivan D.A., Schaumberg D.A. (2007). Impact of dry eye syndrome on vision-related quality of life. Am. J. Ophthalmol..

[9] Li M., Gong L., Sun X., Chapin W.J. (2011). Anxiety and depression in patients with dry eye syndrome. Curr. Eye Res..

[10] Barabino S., Labetoulle M., Rolando M., Messmer E.M. (2016). Understanding symptoms and quality of life in patients with dry eye syndrome. Ocul. Surf..

[11] Aragona P., Giannaccare G., Mencucci R., Rubino P., Cantera E., Rolando M. (2021). Modern approach to the treatment of dry eye disease: A P.I.C.A.S.S.O. board review. Br. J. Ophthalmol..

[12] Aragona P., Rolando M. (2013). Towards a dynamic customised therapy for ocular surface dysfunctions. Br. J. Ophthalmol..

[13] Rolando M., Cantera E., Mencucci R., Rubino P., Aragona P. (2018). The correct diagnosis and therapeutic management of tear dysfunction: Recommendations of the P.I.C.A.S.S.O. board. Int. Ophthalmol..

[14] Aragona P., Giannaccare G., Rolando M. (2022). Special issue “Managing dry eye disease over time: An Italian consensus conference”. J. Clin. Med..

[15] Pill J. (1971). The Delphi method: Substance, context, a critique and an annotated bibliography. Socio-Econ. Plan. Sci..

[16] Rowe G., Wright G., Bolger F. (1991). Delphi: A re-evaluation of research and theory. Technol. Forecast. Soc. Change.

[17] Jones J., Hunter D. (1995). Qualitative research: Consensus methods for medical and health services research. BMJ.

[18] van Setten G., Labetoulle M., Baudouin C., Rolando M. (2016). Evidence of seasonality and effects of psychrometry in dry eye disease. Acta Ophthalmol..

[19] Horwath-Winter J., Berghold A., Schmut O., Floegel I., Solhdju V., Bodner E., Schwantzer G., Haller-Schober E.M. (2003). Evaluation of the clinical course of dry eye syndrome. Arch. Ophthalmol..

[20] Lienert J.P., Tarko L., Uchino M., Christen W.G., Schaumberg D.A. (2016). Long-term natural history of dry eye disease from the patient’s perspective. Ophthalmology.

[21] Ezuddin N.S., Alawa K.A., Galor A. (2015). Therapeutic strategies to treat dry eye in an aging population. Drugs Aging.

[22] Kawashima M., Uchino M., Yokoi N., Uchino Y., Dogru M., Komuro A., Sonomura Y., Kato H., Kinoshita S., Tsubota K. (2016). The association of sleep quality with dry eye disease: The Osaka study. Clin. Ophthalmol..

[23] Wu M., Liu X., Han J., Shao T., Wang Y. (2019). Association between sleep quality, mood status, and ocular surface characteristics in patients with dry eye disease. Cornea.

[24] Li S., Ning K., Zhou J., Guo Y., Zhang H., Zhu Y., Zhang L., Jia C., Chen Y., Sol Reinach P. (2018). Sleep deprivation disrupts the lacrimal system and induces dry eye disease. Exp. Mol. Med..

[25] Kasetsuwan N., Satitpitakul V., Changul T., Jariyakosol S. (2013). Incidence and pattern of dry eye after cataract surgery. PLoS ONE.

[26] Rosenthal P., Borsook D. (2016). Ocular neuropathic pain. Br. J. Ophthalmol..

[27] Kohlhaas M. (1998). Corneal sensation after cataract and refractive surgery. J. Cataract. Refract. Surg..

[28] Khanal S., Tomlinson A., Esakowitz L., Bhatt P., Jones D., Nabili S., Mukerji S. (2008). Changes in corneal sensitivity and tear physiology after phacoemulsification. Ophthalmic. Physiol. Opt..

[29] Epitropoulos A.T., Matossian C., Berdy G.J., Malhotra R.P., Potvin R. (2015). Effect of tear osmolarity on repeatability of keratometry for cataract surgery planning. J. Cataract. Refract. Surg..

[30] Baudouin C., Aragona P., Messmer E.M., Tomlinson A., Calonge M., Boboridis K.G., Akova Y.A., Geerling G., Labetoulle M., Rolando M. (2013). Role of hyperosmolarity in the pathogenesis and management of dry eye disease: Proceedings of the OCEAN group meeting. Ocul. Surf..

[31] Cejková J., Ardan T., Cejka C., Luyckx J. (2011). Favorable effects of trehalose on the development of UVB-mediated antioxidant/pro-oxidant imbalance in the corneal epithelium, proinflammatory cytokine and matrix metalloproteinase induction, and heat shock protein 70 expression. Graefes Arch. Clin. Exp. Ophthalmol..

[32] Luyckx J., Baudouin C. (2011). Trehalose: An intriguing disaccharide with potential for medical application in ophthalmology. Clin. Ophthalmol..

[33] Emanuele E. (2014). Can trehalose prevent neurodegeneration? Insights from experimental studies. Curr. Drug Targets.

[34] Chen W., Zhang X., Liu M., Zhang J., Ye Y., Lin Y., Luyckx J., Qu J. (2009). Trehalose protects against ocular surface disorders in experimental murine dry eye through suppression of apoptosis. Exp. Eye Res..

[35] Aragona P., Colosi P., Rania L., Colosi F., Pisani A., Puzzolo D., Micali A. (2014). Protective effects of trehalose on the corneal epithelial cells. Sci. World J..

[36] Iturriaga G., Suárez R., Nova-Franco B. (2009). Trehalose metabolism: From osmoprotection to signaling. Int. J. Mol. Sci..

[37] Sutu C., Fukuoka H., Afshari N.A. (2016). Mechanisms and management of dry eye in cataract surgery patients. Curr. Opin. Ophthalmol..

[38] Tuft S., Lakhani S. (2008). Medical management of dry eye disease. Dev. Ophthalmol..

[39] Giannaccare G., Scorcia V. (2020). False myths versus medical facts: Ten common misconceptions related to dry eye disease. Biomedicines.

